# The beneficial effects of commensal *E. coli* for colon epithelial cell recovery are related with Formyl peptide receptor 2 (Fpr2) in epithelial cells

**DOI:** 10.1186/s13099-023-00557-w

**Published:** 2023-06-15

**Authors:** Keqiang Chen, John McCulloch, Rodrigo Das Neves, Gisele Rodrigues, Wang-Ting Hsieh, Wanghua Gong, Teizo Yoshimura, Jiaqiang Huang, Colm O’hUigin, Simone Difilippantonio, Matthew McCollum, Georgette Jones, Scott K. Durum, Giorgio Trinchieri, Ji Ming Wang

**Affiliations:** 1 Laboratory of Cancer Innovation, Center for Cancer Research, National Cancer Institute at Frederick, Frederick, MD 21702 USA; 2 Laboratory of Integrative Cancer Immunology, Center for Cancer Research, National Cancer Institute, Bethesda, MD 20892 USA; 3 Animal Health Diagnostic Laboratory, Frederick National Laboratory for Cancer Research, Frederick, MD 21702 USA; 4 Basic Research Program, Leidos Biomedical Research, Inc, Frederick, MD 21702 USA; 5 Department of Pathology and Experimental Medicine, Graduate School of Medicine, Dentistry and Pharmaceutical Sciences, Okayama University, Okayama, 700-8558 Japan; 6 College of Life Sciences, Beijing Jiaotong University, Beijing, 100044 People’s Republic of China; 7 Gnotobiotics Facility, Frederick National Laboratory for Cancer Research, Frederick, MD 21702 USA

**Keywords:** Commensal *E. coli*, *Fpr2*^*−/−*^ mice, Germ-free mice, Colitis, Regeneration

## Abstract

**Background:**

Formyl peptide receptor 2 (Fpr2) plays a crucial role in colon homeostasis and microbiota balance. Commensal *E. coli* is known to promote the regeneration of damaged colon epithelial cells. The aim of the study was to investigate the connection between *E. coli* and Fpr2 in the recovery of colon epithelial cells.

**Results:**

The deficiency of Fpr2 was associated with impaired integrity of the colon mucosa and an imbalance of microbiota, characterized by the enrichment of Proteobacteria in the colon. Two serotypes of *E. coli*, O22:H8 and O91:H21, were identified in the mouse colon through complete genome sequencing. *E. coli* O22:H8 was found to be prevalent in the gut of mice and exhibited lower virulence compared to O91:H21. Germ-free (GF) mice that were pre-orally inoculated with *E. coli* O22:H8 showed reduced susceptibility to chemically induced colitis, increased proliferation of epithelial cells, and improved mouse survival. Following infection with *E. coli* O22:H8, the expression of Fpr2 in colon epithelial cells was upregulated, and the products derived from *E. coli* O22:H8 induced migration and proliferation of colon epithelial cells through Fpr2. Fpr2 deficiency increased susceptibility to chemically induced colitis, delayed the repair of damaged colon epithelial cells, and heightened inflammatory responses. Additionally, the population of *E. coli* was observed to increase in the colons of *Fpr2*^*−/−*^ mice with colitis.

**Conclusion:**

Commensal *E. coli* O22:H8 stimulated the upregulation of Fpr2 expression in colon epithelial cells, and the products from *E. coli* induced migration and proliferation of colon epithelial cells through Fpr2. Fpr2 deficiency led to an increased *E. coli* population in the colon and delayed recovery of damaged colon epithelial cells in mice with colitis. Therefore, Fpr2 is essential for the effects of commensal *E. coli* on colon epithelial cell recovery.

**Supplementary Information:**

The online version contains supplementary material available at 10.1186/s13099-023-00557-w.

## Background

The colonization of the gut by microbes in mammals occurs during and after birth. This process is dynamic and influenced by various factors such as lifestyle, diet, host genotype, antibiotic use, and diseases [1]. These interactions eventually lead to the establishment of diverse bacterial populations that form a symbiotic relationship with the host [2]. The term “dysbiosis” was coined over a century ago to describe the disruption of this symbiosis [3]. Dysbiosis involves the imbalance of beneficial microbial input or signals and the expansion of pathogenic microbes, known as pathobionts. It is believed that dysbiosis can trigger pro-inflammatory responses and immune dysregulation, which are associated with various disease states [4], including inflammatory bowel disease (IBD) [5].

Inflammatory bowel disease (IBD) is characterized by chronic and recurrent inflammation of the intestines and includes two main subtypes: ulcerative colitis (UC) and Crohn's disease (CD). UC primarily involves inflammation of the colonic mucosa throughout the colon, while CD is characterized by transmural ulceration that can occur in any part of the gastrointestinal tract, with the terminal ileum and colon being most commonly affected [6].

The pathogenesis of IBD is complex and not fully understood. Current evidence suggests that dysregulation of immune responses to intestinal flora, as well as interactions between genetic and environmental factors, play a significant role in driving the disease [6, 7]. However, the exact underlying pathogenic mechanisms that lead to IBD development and progression are still not completely elucidated [6, 7]. Further research is necessary to gain a comprehensive understanding of the factors involved in the pathogenesis of IBD.

Formyl peptide receptors (FPRs in humans and Fprs in mice) are members of the G-protein-coupled chemoattractant receptors (GPCRs) family [8]. They play a role in recognizing natural and synthetic ligands and mediating the accumulation of myeloid cells at sites of infection and inflammation [9, 10]. In humans, there are three functional FPRs: FPR1, FPR2, and FPR3 [11, 12], while in mice, there are at least two counterparts: Fpr1 and Fpr2 [13]. FPR2 exhibits a diverse expression pattern and functionality, as it can recognize a wide range of formylated or non-formylated chemotactic agonists derived from pathogens, host cells, synthetic peptides, and small molecules [14–16]. FPR2 has been implicated in various human diseases, including infections, inflammation, and cancer [17, 18].

The use of *Fpr2*^*−/−*^ mice has provided valuable models for studying human diseases, such as allergic airway inflammation [19], lung carcinoma [20], and colon inflammation and cancer [21]. By examining the changes in the colon microbiota of *Fpr2*^*−/−*^ mice, we can gain a deeper understanding of the mechanisms underlying human diseases and develop improved therapeutic strategies. This approach allows for a more comprehensive exploration of the relationship between Fpr2 and the gut microbiota, shedding light on the pathogenesis of various conditions and facilitating the development of targeted treatments.

Mouse Fpr2 exhibits a significant role in maintaining colon homeostasis by being expressed on colon crypt epithelial cells and promoting cell proliferation [8, 21]. This response is triggered by the chemotactic agonist fMLF, which is released by enteric bacteria like *Lactobacillus rhamnosus* GG (LGG) and *E. coli* [21, 22]. In healthy individuals, *E. coli* represents a small fraction, less than 1%, of the gut microbiota [23]. However, in individuals with inflammatory bowel disease (IBD) and in animal models of gut inflammation, *E. coli* becomes more dominant in the gut microbiota [24–26]. *E. coli* strains isolated from individuals with IBD often exhibit adherence and invasive properties, displaying virulence characteristics [27].

In the current study, we identified two serotypes of *E. coli* and established the connection between the commensal *E. coli* serotype O22:H8 and Fpr2. This finding suggests a specific relationship between commensal *E. coli* strain and the Fpr2 receptor, potentially implicating their involvement in the modulation of colon homeostasis and the pathogenesis of intestinal diseases.

## Methods

### Mice

Specific-pathogen-free (SPF) mice: *Fpr2*^*−/−*^ mice were generated as described previously [19]. Cre-loxp strategy [28] was used to deplete mouse Fpr2 gene. *Fpr2*^*−/−*^ mice were backcrossed for at least eight generations to wild type (WT) C57BL/6 mice before using experiments. WT (*Fpr2*^+/+^) and *Fpr2*^−/−^ litter mates were generated by mating pairs of male and female heterozygous (*Fpr2*^+/−^) mice. All mice were maintained under SPF environment in the facility of Frederick National Laboratory for Cancer Research (Frederick, MD). Mice were allowed for free access to standard laboratory chow/tap water. All animals were housed in an air-conditioned room with controlled temperature (22 ± 1 °C), humidity (65–70%), and day/night cycle (12 h light, 12 h dark). The mice used in the experiments were male and 2–3 months old (except in the experiments to detect crypt length of colons at different mouse ages).

Germ-free (GF) mice: The embryos were obtained from pregnant female C57BL/6 wild type (WT) mice under sterile conditions then transplanted into a germ-free mother mouse to obtain GF WT mice. GF mice were maintained in a sterile environment completely devoid of microorganisms in Gnotobiotic Facility, Frederick National Laboratory for Cancer Research, Frederick, MD. The GF mice used in the experiments were 2–3 months old, males and all experiments were performed under sterile conditions.

### Cell culture

CT26 mouse colon carcinoma cell line was maintained in Dulbecco's modified Eagle's medium (DMEM) (Gibco-Invitrogen) containing 10% FBS (HyClone Laboratories, Logan, UT, USA) and 1% penicillin/streptomycin. CT26 cells were cultured in a humidified 37˚C incubator with 5% CO2.

### Design of animal experiments

1. For measuring the effect of Fpr2 deficiency on colon crypt length, male WT and *Fpr2*^*−/−*^ mice (4–9 mice per group) were euthanized at ages of 1, 15, 30, and 90 days. The colons were harvested and used to examine the crypt length. The colon sections of WT and *Fpr2*^*−/−*^ mice at age of 60 days were further used to examine the levels of Muc2, cathelin-related antimicrobial peptide (CRAMP), β-Defensin, PAS^+^ goblet cells and Ki67.
2. Evaluation of the composition of gut microbiome in naïve mice. Male WT and *Fpr2*^−/−^ mice were co-housed (2–3 WT mice plus 2–3 *Fpr2*^*−/−*^ mice/cage) for 4 weeks after weaning, followed by separation (4–5 WT mice/cage or 4–5 *Fpr2*^*−/−*^ mice/cage) for an additional 6 weeks. Subsequently, each mouse was individually housed in a separate cage to collect fresh fecal pellets. The fecal pellets were collected in tubes, with one tube assigned to each mouse. These fecal samples were collected for the purpose of analyzing the microbiota through 16S rRNA gene sequencing.

For analysis of fecal microbiota by 16S rRNA gene sequencing, fecal DNA was prepared using the DNA Stool Mini Kit (QIAGEN) for sequencing. Briefly, the V4 fragment of 16S rDNA was amplified by PCR using primers 515F: 5′-GTGCCAGCMGCCGCGGTAA-3′ and 806R: 5′-GGACTACHVGGGTWTCTAAT-3′ flanked by p5 and p7 Illumina Sequencing adaptors (p5 and p7), barcodes (i5 and i7), pad (to optimize melting temperature), and a link sequence. PCR products were purified and normalized using a SequalPrep Normalization Plate Kit (Invitrogen). Sequencing was performed using Mothur v.1.30.0. as described in the MiSeq 16S standard operating procedure protocol. All sequencing was performed using the National Institutes of Health Biowulf Cluster. The bioinformatics analysis of 16S rRNA gene sequencing data of microbiota was conducted using software USEARCH version 9.2.64 and QIIME version 1.9.1. Paired-end reads were merged, and stringent quality filtering was performed to remove low-quality reads. The remaining reads were de-replicated and clustered into operational taxonomic units (OTUs) with 97% sequence identity using the UPARSE algorithm. OTU assignment and creation of an OTU table were done using the usearch_global command. Taxonomy was assigned using BLASTn searches against the SILVA ribosomal RNA gene database. OTUs with fewer than 10 sequences and no BLASTn hit were removed as quality control. The OTU tables were further processed by rarefying to the sample with the lowest number of sequences, with a threshold of > 10,000 sequences. Statistical analysis was performed using ANOVA and *P* values were corrected for multiple comparisons using the q-value test (0.1) [29]. The results of fecal bacterial population were represented as a Heat-map.

3. For DSS-induced colitis, male WT and *Fpr2*^*−/−*^ mice (10–12 mice per group) were administered 3% dextran sulfate sodium (DSS) (M.W. = 36,000–50,000, MP Biomedicals, LLC) in their drinking water for a duration of 5 days. Following this, they were provided with normal water for a period of 7 days to observe mouse survival. Furthermore, additional groups of WT and *Fpr2*^*−/−*^ mice was administered 5% DSS for 5 days, after which they were euthanized. The colons from these mice were harvested and sectioned for the assessment of colon mucosal damage.
4. To evaluate the recovery of colon epithelial cells, a group of male WT mice and a group of *Fpr2*^*−/−*^ mice (6 mice per group) were subjected to a treatment with 5% DSS for a duration of 3 days, followed by a period of normal water consumption for 4 days. After this treatment period, the mice were euthanized, and their colons were harvested and sectioned for further analysis.

The levels of Ki67, which is a marker of cell proliferation, were examined to assess the regenerative capacity of the colon epithelial cells. Additionally, the presence of PAS + goblet cells, which produce mucus, Muc2 (a key mucin protein), and IL-1β (an inflammatory cytokine), were also evaluated. These measurements were performed to gain insights into the recovery and functionality of colon epithelial cells in both WT and *Fpr2*^*−/−*^ mice following the DSS treatment and subsequent recovery period.

5. For quantification of colony forming units (CFU) of *E. coli* in feces, the mice given with 5% DSS for 5 days were euthanized and the colon with cecum (avoid fecal leakage) were harvested. Feces (50–70 mg per mouse) were used for culture of *E. coli* on Violet Red Bile Lactose agar (VRBL, EMD Millipore Corporation) to compare the number of *E.coli* between WT and *Fpr2*^*−/−*^ mice. Single colonies on VRBL were harvested and amplificated in LB Broth, then extracted for *E. coli* DNA with the DNeasy UltraClean Microbial Kit (GIAGEN, MD). *E. coli* were identified with PCR and 16S rRNA gene sequencing.

### Conventional PCR for genes of 16S rRNA and LpfA of *E. coli*

For the amplification of *E. coli* 16S rRNA and LpfA gene, a conventional PCR method was employed. Purified *E. coli* DNA was quantified using an NP-1000 Spectrophotometer (Thermo, MD) and adjusted to the same concentration for each sample, which was 1 µg/µl. For the amplification of *E. coli* 16S rRNA, the following primer sequences were used: primers 5′-TGG CTC AGG ACG AAC GCT GGC GGC-3′ (sense) and 5′-CCT ACT GCT GCC TCC CGT AGG AGT-3′ (antisense) were designed to yield a 348-bp product. The PCR amplification condition consisted of an initial denaturation step at 95 °C for 5 min, followed by 30 cycles of denaturation at 95 °C for 45 s, annealing at 58 °C for 1 min, extension at 72 °C for 45 s, and a final extension step at 72 °C for 10 min. The PCR products were separated and visualized on 1.5% agarose gels through electrophoresis, followed by staining with ethidium bromide. Similarly, for the amplification of *E. coli* LpfA gene, the following primer sequences were used: Sense primer: 5′-AGTTGGTGATAAATCACCAT-3′, Antisense primer: 5′-GTGCTGGATTCACCACTATTCATCG-3′. These primers were designed to yield a 222-bp product specific to the LpfA gene. The PCR amplification condition and product visualization were the same as mentioned above.

### Histologic and immunohistochemical staining

To prepare the colon tissues for analysis, they were embedded in Optimal cutting temperature compound (OCT) and frozen. The frozen tissues were then sectioned into slices that were 10-μm thick. These sections were subsequently fixed in 8% neutral buffered formalin for 30 min to preserve their structure. After fixation, the sections were washed three times with distilled water (ddH2O) to remove any residual formalin. Hematoxylin and eosin (H&E) staining was performed on the sections to visualize the tissue morphology. The H&E-stained sections of the colon tissue were observed using an Olympus microscope equipped with a DP80 camera. Images of the crypts were captured for further analysis. The length of the crypts was measured using ImageJ (a Java-based image processing program developed at the National Institutes of Health).

For the assessment of colon mucosal damage, histopathological scoring was performed. The scoring system typically involves assigning grades ranging from 0 to 5 based on several criteria, including the extent of colon tissue affected, the extent of crypt damage, and the quantity and dimension of inflammatory cell infiltration [30]. The scoring system provides a standardized way to evaluate the severity of colon mucosal damage. In this study, 4–8 mice were used for each mouse group, and the histopathological scoring was performed on multiple sections from each mouse to obtain representative data.

For detection of bacteria attaching to colon mucosa, Bacterial Gram Staining Kit (Abcam, MA) was used following the manufacturer’s protocol. Sections were dehydrated in absolute alcohol, cleared in xylene, and then mounted in a synthetic resin.

To detect goblet cells in the colon crypts, the slides were stained with the Alcian Blue Periodic Acid-Schiff (PAS) Stain Kit. 23–25 crypts from 4 mice per mouse group were analyzed for the quantification of PAS + cells.

### Immunofluorescence staining

To perform immunofluorescence staining on the colon tissue sections, the frozen sections embedded in OCT were fixed in 8% neutral buffered formalin for 30 min. After fixation, the sections were washed three times with distilled water (ddH2O) to remove residual formalin. For the staining process, primary antibodies specific to the target proteins of interest, such as anti-mouse Muc2 (ab76774, Abcam), CRAMP (sc-66843, Santa Cruz), or β-Defensin 2 (ab203077, Abcam), Ki67 (ab16667, Abcam), IL-1β (AF-401-NA, R&D) antibodies and anti *E. coli* antibody **(**ab25823**,** Abcam**),** were applied to the sections. Following the primary antibody incubation, secondary antibodies conjugated with biotin (ab6720, ab208000, ab207997, ab207996, Abcam and BAF109, R&D), were applied to the sections followed with streptavidin-FITC (405202, BioLegend) or PE (405204, BioLegend), allowing for the visualization of the target proteins. The 6-diamidino-2-phenylindole (DAPI) was used to stain the cell nuclei. The fluorescence intensity of all immunofluorescence staining was measured by ImageJ.

### Fluorescence in situ hybridization (FISH)

Detection of bacteria on colon mucosa was performed with freshly frozen, OCT-embedded, and sectioned slides (10-μm thick). The slides were fixed in 4% neutral buffered formalin for 5 min, washed twice with 1 × PBS (DEPC treated) and pre-hybridized with 200 μl 3% BSA at 37 °C for 2 h. The sections were placed in the Slide Griddle (Model SG96P, MJ Research, Inc., MA) and in 96 V PTC Thermal Cyclers (MJ Research, Inc., MA) and heated to denature at 84 °C for 5 min, 37 °C for 3 min before adding FISH probe (0.1–0.4 μm in Hybridization buffer) and incubated overnight at 40 °C. The slides were washed with washing solution (2 × SSC/0.1% Tween 20) for 15 min at RT followed by treatment with 75% and 100% ethanol, then air dried. DAPI-Antifade solution was added in the dark for 10 min to stain nuclei. The sections were then mounted in Resolve (Thermo Scientific, MI, USA) and stored at 4 °C. The probes conjugated to CY3 were used to detect bacteria adhering to colon epithelial cells. The universal bacterial EUB338 FISH Probe (5′-CTGCCTCCCGTAGGAGT-3′) (Creative Bioarray, NY) conjugated with CY3 was used to detect total bacteria. A ‘non-sense’ probe (5′-CGACGGAGGGCATCCTCA-3′) conjugated with CY3 was used as a negative control for EUB338. EC1531 FISH Probe (5′CACCGTAGTGCCTCGTCATCA-3′) conjugated with CY3 (Integrated DNA Technologies) was used to detect *E. coli*.

### Enzyme-linked immunosorbent assay (ELISA)

To measure fecal Muc2, β-definsin-2 (DEFb2), cathelin-related antimicrobial peptide (CRAMP), and Lipopolysaccharides (LPS), freshly harvested feces were homogenized in PBS containing 1% NaN_3_, 20 mM dithiothreitol, and a protease inhibitor mixture (P8340; Sigma, 1:200 dilution) [31]. Fecal suspensions were centrifuged at 15,000 RPM for 10 min at 4 °C. The supernatants were collected and inactivated at 60 °C for 30 min, and their protein concentrations were determined using a DC Protein Assay Kit (Bio-Rad). The concentrations of Muc2, DEFb2, CRAMP and LPS in the feces were determined with ELISA specific to Muc2 (Cloud-Clone Corp., TX, USA), DEFb2 (Cloud-Clone Corp., TX, USA), CRAMP (MybioSource, CA, USA), and LPS (MybioSource, CA, USA) respectively. The Muc2 concentration was expressed in ng per 1 mg (mg) protein of stool. The concentrations of DEFb2 and CRAMP in the feces were expressed in pg per 1 mg (mg) protein of stool. The concentrations of LPS in the feces were expressed ng/g stool.

To measure IL-1β in the mucosa, colons were washed with PBS containing antibiotics (penicillin/streptomycin) and the distal 3 cm were isolated and further cut into1–2 mm sections. Colon sections were covered with RPMI media (1 ml), containing 1% FBS and penicillin/streptomycin overnight in a humidified 37˚C incubator with 5% CO2. Cell-free supernatants were harvested and their protein concentrations were determined using a DC Protein Assay Kit (Bio-Rad) [32]. The samples were stored at − 80 °C for ELISA assay. IL-1β concentrations were measured by ELISA (Thermo Fisher). LPS in serum was measured with mouse LPS ELISA Kit (MybioSource, CA) and the concentration of LPS in serum were expressed in ng/ml.

### Identification of *E. coli*

Single colonies of *E. coli* on Violet Red Bile Lactose agar were harvested and smeared onto slides and stained with Gram stain Kit (abcam, MA) and examined with Fluorescence in situ hybridization (FISH) with EC1531 probe and PCR to identify *E. coli*. The colonies of *E. coli* identified were selected for 16S rRNA sequencing. Single colonies with different 16S rRNA sequences were then used for whole genome sequencing. *E. coli* serotypes O22H8 and O91H21 were defined based on databases and kept in − 80 °C for further use.

### Adhesion of colon epithelial cells by *E*. *coli*

Mouse epithelial CT26 cells (1 × 10^6^ cells/ml) were cultured with *E. coli* (1 × 10^7^ CFU/ml) at a multiplicity of 10 bacteria per cell in 14 ml polypropylene round-bottom tubes (FALCON, 352059) at 37 °C with shaking at 60 RPM for 2 h. The cells were smeared onto slides, stained with a three-step staining kit (Thermo Scientific Richard-Allan Scientific), and observed under an Olympus microscope with a DP80 camera.

### *E. coli-*induced death of colon epithelial cells

One million CT26 cells were seeded in 35 mm dishes with 14 mm coverslips in the bottom and co-cultured with *E. coli* O22:H8 and O91:H21 at a multiplicity of 10 bacteria per cell for 6 h at 37 °C. The cells were washed two times with PBS, fixed with 4% neutral buffered formalin for 5 min and then stained with Live/Dead Viability/Cytotoxicity Kit for mammalian cells. The slides were observed under an Olympus microscope with a DP80 camera.

### Chemotaxis assays

Cells were performed with 48-well chemotaxis chambers and polycarbonate filters (8-µm pore size) (NeuroProbe, Cabin John, MD). The results are expressed as the mean ± S.D. of the chemotaxis index, which represents the fold increase in the number of migrated cells, counted in three high power fields (×400), in response to chemoattractants over spontaneous cell migration (to control medium).

### Testing *E. coli* in germ-free (GF) mice

GF mice were orally inoculated with *E. coli* O22:H8 or O91:H21 (2 × 10^8^ CFU live *E. coli* per mouse). Five days after inoculation, all mice were sacrificed, or the mice were given 3% DSS for 4 days and colons were harvested.

### Ethical statement

Animal protocols were in accordance with the recommendations of the US NIH Guide for the Care and Use of Laboratory Animals (National Academies Press, 2011) and were approved by the Frederick National Laboratory for Cancer Research Animal Care and Use Committee (ASP No. 21-464).

### Statistical analysis

All experiments were performed at least three times with reproducible results. Statistical analyses, unless specified, were performed by GraphPad Prism 9 (GraphPad Software, San Diego, CA). A *P* value of < 0.05 was considered statistically significant.

## Results

### Impaired colon mucosal barrier in ***Fpr2***^***−/−***^ mice

**Fig. 1:**
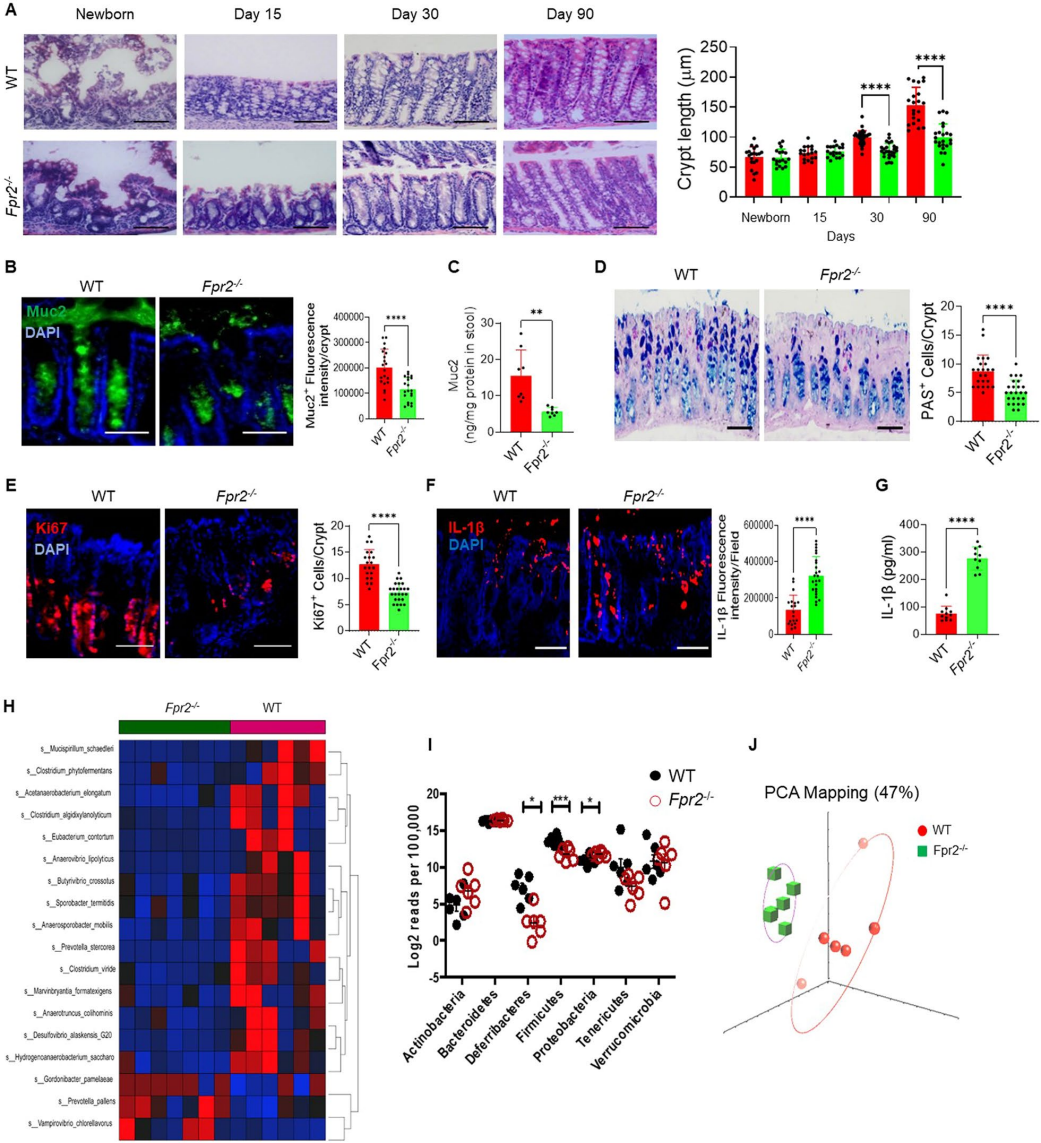
Impaired colon mucosal barrier in *Fpr2*^*−/−*^ mice. **A** Shortened colon crypts in *Fpr2*^*−/−*^ mice at different ages. H&E staining. Scale bar = 100 μm. Right: Quantitation of crypt length of colons at different mouse ages. n = 4–8 mice/group. ****P < 0.0001. **B** Reduced Muc2 production in colon crypts of *Fpr2*^*−/−*^ mice. Green: Muc2, Blue: Nuclei. Scale bar = 50 μm. Right: Quantitation of Muc2^+^ fluorescence intensity/crypt, n = 18–20 crypts from 6 mice/group, ****P < 0.0001. **C** Reduced level of Muc2 in the feces of *Fpr2*^*−/−*^ mice. The Muc2 concentration in the feces was expressed as ng/1 mg protein in feces. n = 8 mice/group, **P < 0.01. **D** Reduced number of goblet cells in the colon of *Fpr2*^*−/−*^ mice. An alcian blue stain. Scale bar = 50 μm. Right panel Quantitation of PAS^+^ goblet cells per crypt, n = 23–25 crypts from 4 mice/group. ****P < 0.0001. **E** Reduced number of Ki67^+^ cells in the colon of *Fpr2*^*−/−*^ mice. Red: Ki67^+^ cells, Blue: Nuclei. Scale bar = 50 μm. Right: Quantitation of Ki67^+^ cells per crypt, n = 20–25 crypts from 4 mice/group. ****P < 0.0001. **F** Increased production of IL-1β in the colon mucosa of *Fpr2*^*−/−*^ mice. Red: IL-1β, Blue: Neuclei. Scale bar = 50 μm. **Right**: Quantitative IL-1β + fluorescence intensity, n = 19–24 crypts from 4 mice/group. ****P < 0.0001. **G** Increased production of IL-1β in the colon mucosa of *Fpr2*^*−/−*^ mice measured by ELISA. n = 10–11 mice/group, ****P < 0.0001. **H-J** WT (*Fpr2*^+/+^) and *Fpr2*^−/−^ mice were co-housed for 4 weeks after weaning, followed by separation for an additional 6 weeks. Fecal pellets were collected to analyze microbiota by 16S rRNA sequencing. **H** Heat map showing 18 bacterial strains at the phylum levels in the feces. **I** Quantitation of microbiota in the feces of WT and *Fpr2*^−/−^ mice, showing significantly reduced *Firmicutes* and *Deferribacteres* but increased *Proteobacteria* in *Fpr2*^−/−^ mouse microbiota. *P < 0.05, ***P < 0.001. **J** The principal coordinate analysis (PCA) indicating that the abundance of bacteria in the gut of *Fpr2*^*−/−*^ mice is different from WT mice.

We previously demonstrated the shortening of colonic crypt length in adult *Fpr2*^*−/−*^ mice and suggested that the interaction of Fpr2 with commensal bacteria is critical for colonic tissue homeostasis [21]. Here, we examined the colonic crypt length of WT and *Fpr2*^*−/−*^ mice at different ages. Interestingly, there was no difference in the colonic crypt length between WT and *Fpr2*^*−/−*^ mice up to 30 days after birth (Fig. 1A), suggesting that the shortening of colon crypts in *Fpr2*^*−/−*^ mice is “extrinsic” rather than “intrinsic”. This abnormal development of colon crypts in *Fpr2*^*−/−*^ mice was associated with reduced production of Muc2 (Fig. 1B, C), and cathelin-related antimicrobial peptide (CRAMP) (Additional file 1: Fig. S1A, B), β-defensin-2 (Additional file 1: Fig. S1C, D) in colon mucosa and feces. The importance of Fpr2 in the barrier integrity and anti-microbial defense in mouse colon was further supported by the findings that the numbers of goblet cells (Fig. 1D) and Ki67^+^ proliferating epithelial cells (Fig. 1E) were also reduced in the colon crypts of *Fpr2*^*−/−*^ mice. The production of pro-inflammatory cytokines IL-1β was increased in the colon mucosa of *Fpr2*^*−/−*^ mice as compared to WT mice (Fig. 1F–G).

In summary, our findings highlight the critical role of Fpr2 in maintaining the integrity of the colon mucosal barrier. The absence of Fpr2 in *Fpr2*^*−/−*^ mice leads to abnormal development of colon crypts, reduced production of protective molecules, impaired barrier function, and increased inflammation.

### Dysbiosis in the colon of naïve *Fpr2*^*−/−*^ mice

Fpr2 can be activated by several ligands derived from bacteria and host cells [9, 10]. To identify the effect of Fpr2 expressed on the colon epithelia on gut microbiota, we examined the composition of gut bacteria and found significant differences (q < 0.1) in at least 18 bacterial strains at phylum levels in the feces between *Fpr2*^−/−^ and WT littermates as shown by a heat map (Fig. 1H). *Fpr2*^*−/−*^ mice exhibited reduced population of Deferribacteres and Firmicutes, while Proteobacteria were increased in the feces as compared to WT littermates (Fig. 1I). Principal coordinate analysis (PCA) indicated that the abundance of bacteria in the gut of *Fpr2*^*−/−*^ mice differed from WT mice (Fig. 1J). These results highlight the importance of Fpr2 in maintaining the integrity of the colon mucosal barrier and the balance of gut microbiota.

### Identification of *E. coli* serotypes O22:H8 and O91:H21 in mouse feces

**Fig. 2:**
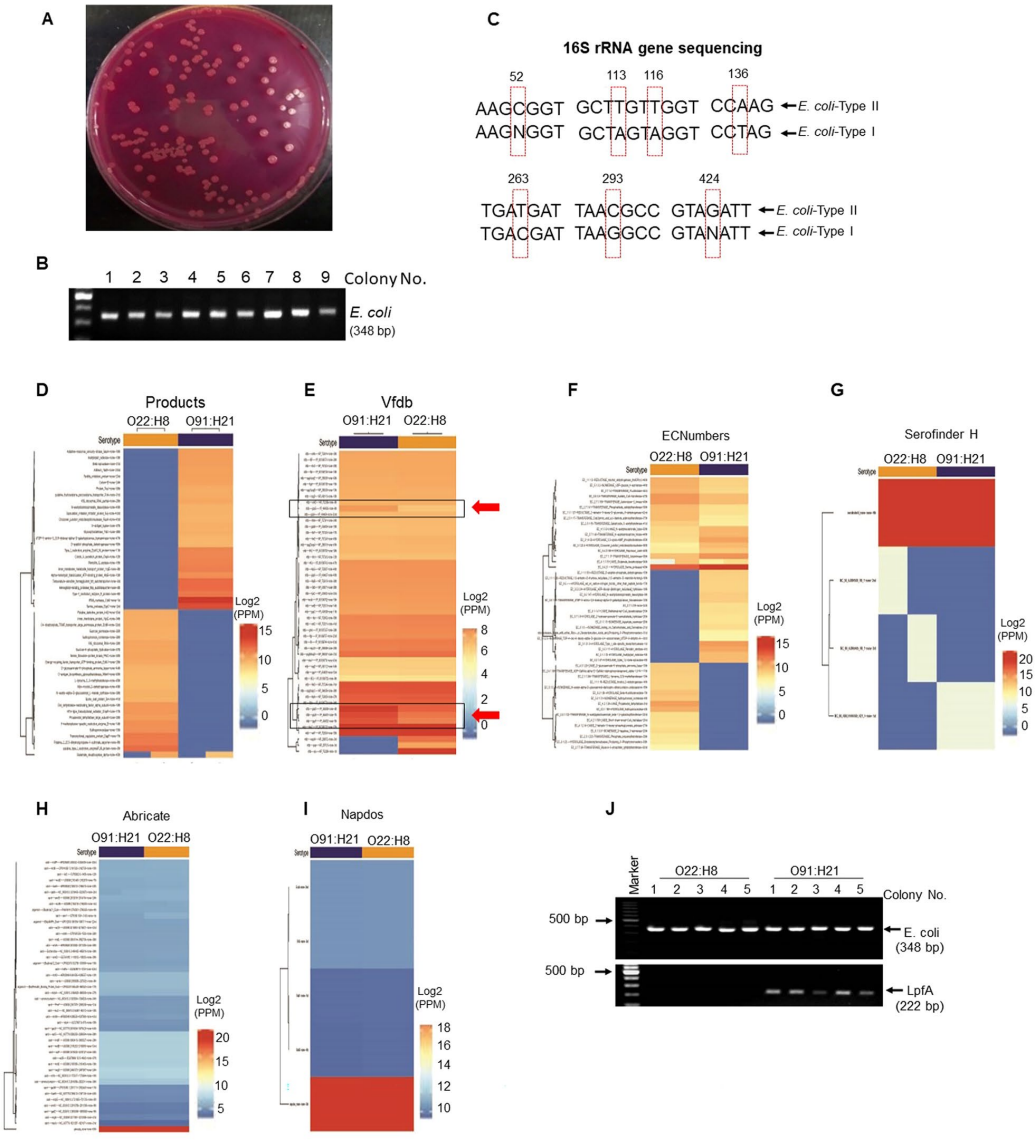
Identification of *E. coli* serotypes O22:H8 and O91:H21 in mouse feces. **A** *E. coli* in mouse feces was isolated by Violet Red Bile Lactose agar (VRVL). **B** PCR analysis confirmed that the bacteria isolated from the mouse feces were *E. coli*. **C** 16S rRNA sequencing for *E. coli*. *E. coli*-Type II showed differences in nucleotide positions 52, 113, 116, 136, 263, 293, and 424 of the 16S rRNA sequence from *E. coli*-Type I. **D–I** Whole genome sequencing of *E. coli* showing relative abundance heatmaps of genes in the serotypes O22:H8 and O91:H21. **D** products. **E** virulence factors. **F** ECNumber: Enzyme Commission number, the Enzyme nomenclature database. **G** Serofinder H: A FASTA database of specific O-antigen processing system genes for O typing and flagellin genes for H typing publicly available Web tools hosted by the Center for Genomic Epidemiology (CGE). **H** Abricate: a database for mass screening of contigs for antimicrobial resistance or virulence genes bundled with multiple databases: NCBI, CARD, ARG-ANNOT, Resfinder, MEGARES, EcOH, PlasmidFinder, *E. coli*_VF and VFDB. **I.** Napdos: a bioinformatic tool for the rapid detection and analysis of secondary metabolite genes. This tool is designed to detect and extract C- and KS- domains from DNA or amino acid sequence data, including PCR amplicon products, individual genes, whole genomes, and metagenomic data sets. **J.** PCR analysis showing higher levels of Long Polar Fimbriae Type I (LpfA) contained in *E. coli* O91:H21

We next isolated *E. coli* from the feces of naïve, DSS alone and AOM-DSS treated mice by VRBL agar (Fig. 2A), a selective agar media for *Enterobacteriace*. After confirming *E. coli* by PCR (Fig. 2B), we further examined 16S rRNA sequences of these *E. coli* colonies (Additional file 1: Fig. S2A, B). Most of the colonies shared similar 16S rRNA sequences (9/10), while a small number of colonies (1/10) exhibited differences in nucleotides #52, 113, 116, 136, 263, 293 and 424 (Fig. 2C and Additional file 1: Fig. S3), which were isolated from DSS-treated *Fpr2*^*−/−*^ mice. Both types of *E. coli* displayed similar Gram-negative (Additional file 1: Fig. S4A), rod-shaped morphology and positive staining with the *E. coli* probe EC1531 [33] (Additional file 1: Fig. S4B). PCR for *E. coli* did not show any differences in the pattern of products (Additional file 1: Fig. S4C). Whole genome sequencing revealed O22:H8 as the serotype for most of the colonies (Type I), while O91:H21 (Type II) was identified as the strain with nucleotide alterations (Fig. 2D–I).

Virulence Factor Database (Vfdb) [34] revealed three putative virulence factor genes present in *E. coli* O91:H21, but not O22:H8 (Fig. 2E), coding for vfdb|gspD|YP_404600, 404601 and 404602 that belong to a “General Secretion Pathway Protein” produced by a generic Type II secretion system (https://www.ebi.ac.uk/QuickGO/term/GO:0015628). In addition, the gene for Long Polar Fimbriae Type I (lpfA) (Fig. 2J), a recognized marker for virulent isolates of pathogenic *E.* *coli* [35, 36], is more readily amplified by PCR in *E.* *coli* O91:H21 than in the O22:H8 strain.

### More virulence of *E. coli* O91:H21 than O22:H8

**Fig. 3:**
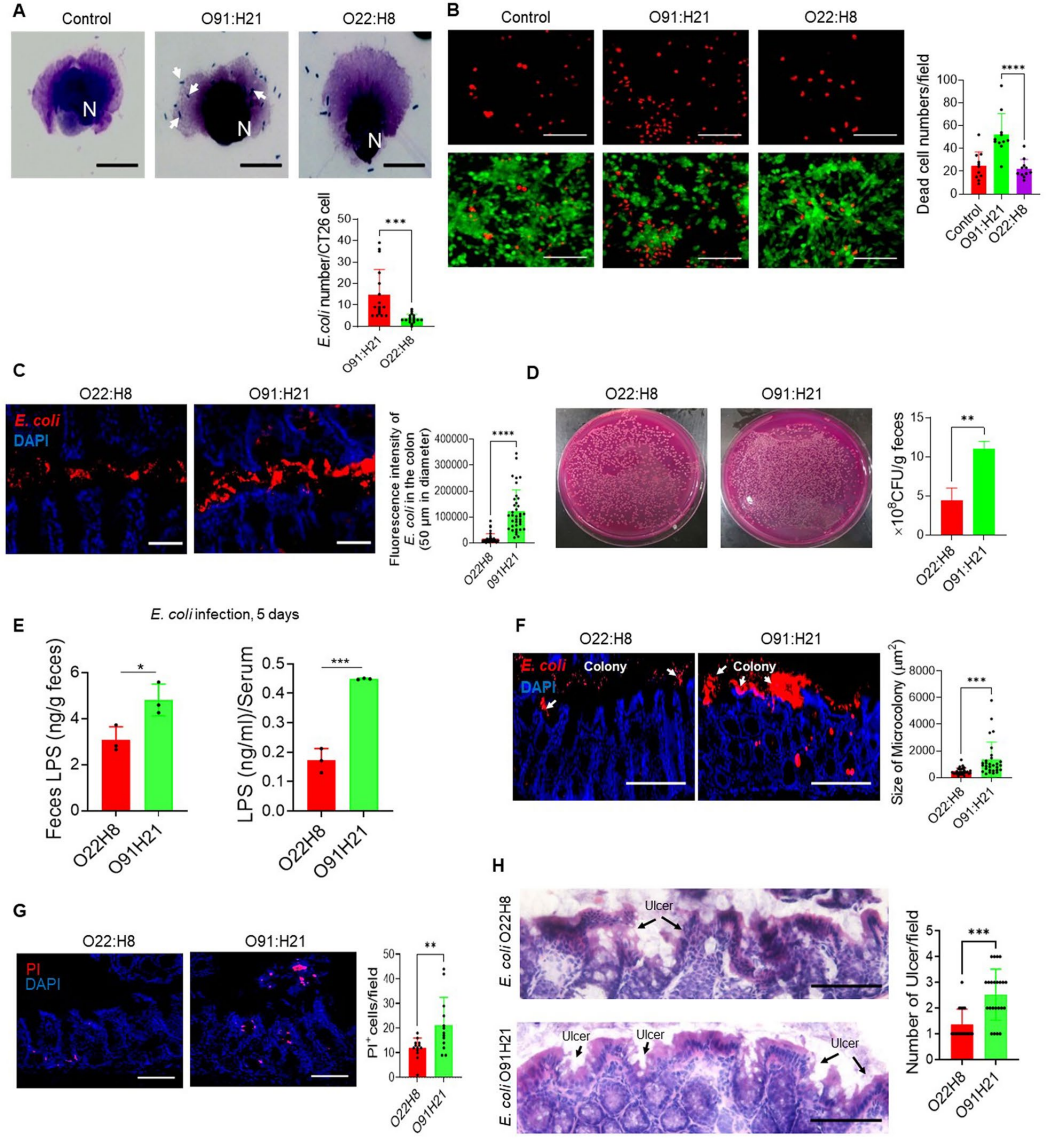
*E. coli* O91:H21 exhibits greater virulence compared to O22:H8. **A** Increased adhesion of *E. coli* O91:H21 to colon epithelial cells**.** CT26 cells: bacteria = 1: 10. Scale bar = 20 μm. N: Nuclei, White arrows: Bacteria surrounding or attaching CT26 cells. Lower: Quantitative bacterial counts adhering to one CT26 cell. n = 27–29 cells/group. ***P < 0.001. **B** More cell death induced by *E. coli* O91:H21 infection. CT26 cells: bacteria = 1:10, Green: live cells; Red: dead cells. Scale bar = 200 µm. Right: Quantitation of dead CT26 cell counts/field. n = 11 fields/group, *P < 0.05. **C** Increased *E. coli* O91:H21 in the colon lumen of GF mice. Red: *E. coli,* Blue: Nuclei. Scale bar = 50 μm. Right: Quantitation of *E. coli*^+^ fluorescence intensity in colon lumen (50 µm in diameter) of GF mice infected with *E. coli* O91:H21 or *E. coli* O22:H8. n = 37 areas (50 µm in diameter) from 4 mice per group, ****P < 0.0001. **D** Increased CFU of *E. coli* O91:H21 in the feces of GF mice. Right: Quantitative CFU (Log10) per g stool from GF mice infected with *E. coli* O91:H21 as compared to mice infected with *E. coli* O22:H8, n = 4 mice/group, **P < 0.01. **E** Increased levels of LPS in the feces and serum of GF mice infected with *E. coli* O91:H21. n = 3 mice/group, *P < 0.05, ***P < 0.001. **F** *E. coli* O91:H21 formed larger colonies on the colon mucosa of GF mice. Red: *E. coli* colonies; Blue: Nuclei. Scale bar = 100 μm. Right: Quantitative *E. coli* colonies in the colon of GF mice infected with *E. coli* O91:H21 or *E. coli* O22:H8. n = 29 colonies from 3 mice per group, ***P < 0.001. **G** Increased colon epithelial cell death in *E. coli* O91:H21 inoculated GF mice. Red: PI + cells in colon mucosa, Blue: Nuclei. Scale bar = 50 μm. Right: Quantitative PI + cells/field. n = 11–13 fields from 3 mice per group. **P < 0.01. **H** Increased ulcer formation in the colon mucosa of GF mice infected with *E. coli* O91:H21. Scale bar = 100 µm. Black arrows: Ulcer. Right: Quantitative number of ulcer in the colon mucosa. n = 17–23 fields from 5 mice/group. ***P < 0.001

In vitro, *E. coli* O91:H21 demonstrated a significantly higher level of adhesion to CT26 mouse colon epithelial cells (Fig. 3A) and caused more extensive cell death (Fig. 3B) as compared to *E. coli* O22:H8. In vivo, a higher number of *E. coli* O91:H21 were detected in the colon lumen and feces of germ-free (GF) mice after oral inoculation with either *E. coli* O22:H8 or O91:H21 for 5 days (Fig. 3C, D). The levels of LPS were significantly elevated in the feces and serum of GF mice inoculated with *E. coli* O91:H21 compared to O22:H8 (Fig. 3E). *E. coli* O91:H21 colonies observed on the surface of the colon mucosa exhibited increased growth (Fig. 3F) and caused more severe damage to the colon epithelial cells (Fig. 3G), leading to a greater number of ulcers in the colon mucosa compared to O22:H8 (Fig. 3H). These findings suggested that *E. coli* O22:H8 is a commensal strain, whereas *E. coli* O91:H21 may possess pathogenic characteristics.

### Fpr2 is required for *E. coli* to promote the colon epithelial cell regeneration

**Fig. 4:**
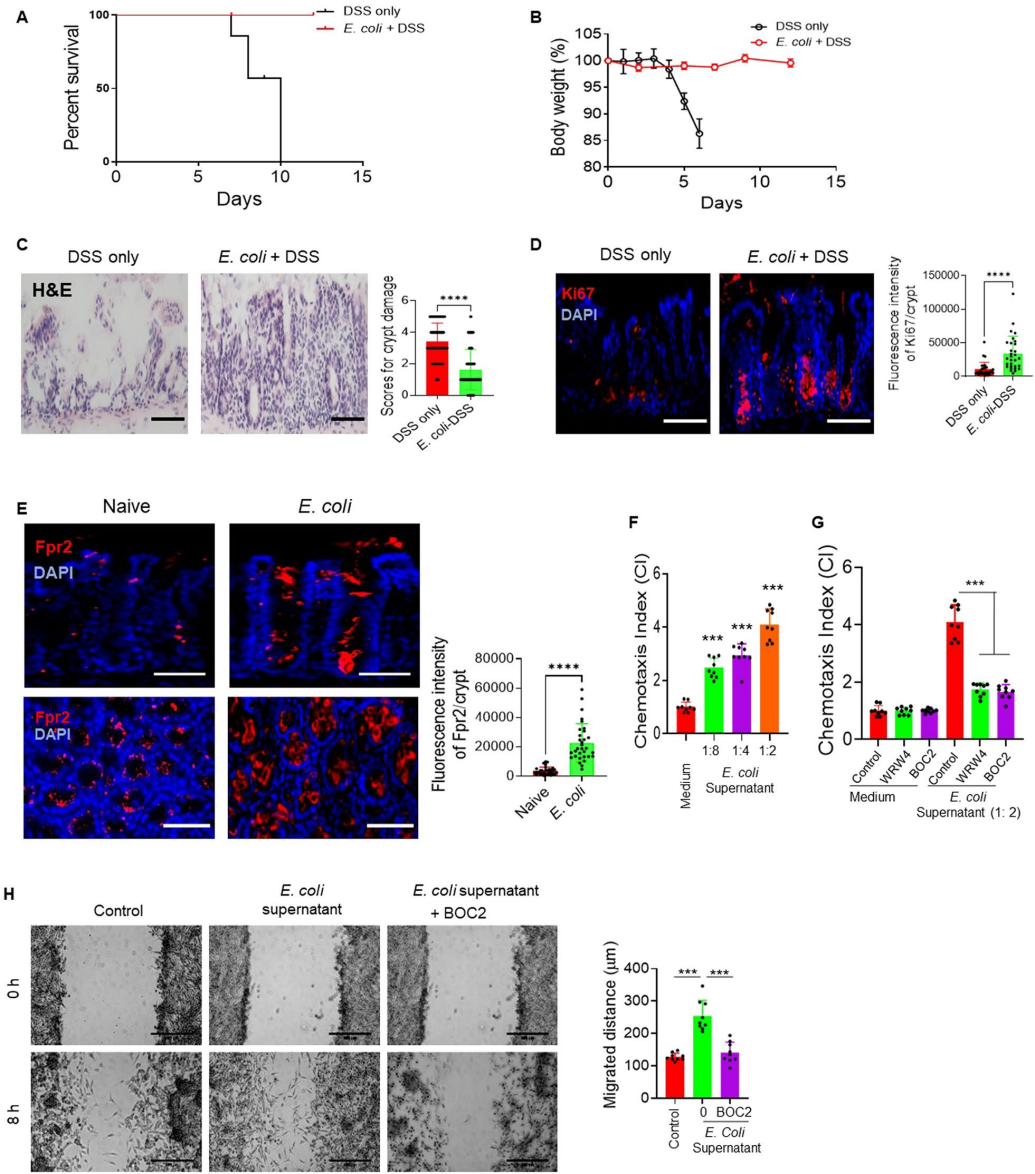
Fpr2 is required for *E. coli* to promote the colon epithelial cell regeneration. **A**, **B** The germ-free (GF) mice pre-orally inoculated with *E. coli* increased mouse survival (**A**) and reduced the loss of body weight (**B**) after challenged with DSS. **C.** Reduced crypt damage in the colon of GF mice pre-orally inoculated with *E. coli.* Scale bar = 50 µm. Right: Quantitative scores for crypt damage. n = 99–122 crypts from 4 mice per group, ****P < 0.0001. **D** Increased number of Ki67 + cells in the colon mucosa of GF mice pre-orally inoculated with *E. coli.* Red: Ki67 + cells, Blue: Nuclei. Scale bar = 50 µm. Right: Quantitative Ki67 + fluorescence intensity/crypt. n = 30 crypts from 4 mice per group, ****P < 0.0001. **E** Upregulation of Fpr2 expression by colon epithelial cells with *E. coli* infection in GF wild type (WT) mice. Upper: Scale bar = 30 µm, lower: Scale bar = 40 µm. Right: Quantitative Fpr2 + fluorescence intensity/crypt. n = 32 crypts from 4 mice/group, ****P < 0.0001. **F** *E. coli* supernatant induced CT26 cell migration. n = 3 mice/group, ***P < 0.001. **G** *E. coli* supernatant induced CT26 cell migration can be inhibited by the *Fpr2* antagonist WRW4 (5 µg/ml) and *Fpr1/Fpr2* antagonist BOC2 (5 µg/ml). n = 3 per group, ***P < 0.001. The results of **F** and **G** are expressed as the mean ± S.D. of the chemotaxis index (CI). **H** *E. coli* supernatant-enhanced closure of CT26 cell monolayer wound was attenuated by *Fpr1/Fpr2* antagonist BOC2 (5 µg/ml). Scale bar = 40 µm. **Right**: Quantitative CT26 cells migration distance (µm) induced by *E. coli* supernatant. BOC2 (5 µg/ml). n = 8 fields/group, ***P < 0.001

To elucidate the role of commensal *E. coli* and its relationship with Fpr2, GF mice were pre-orally inoculated with low levels of *E. coli.* After 5 days, these mice were given with 3% DSS in their drinking water for 5 days, followed by normal water for 7 days. The GF mice that were pre-orally inoculated with *E. coli* exhibited increased survival rates after DSS treatment (Fig. 4A) and showed reduced body weight loss (Fig. 4B) compared to mice subjected to DSS treatment only. Histologically, the GF mice pre-orally inoculated with *E. coli* displayed a significantly reduction in colon mucosal damage (Fig. 4C) and an increased fluorescence intensity of Ki67 + cells in the colon crypts (Fig. 4D).

Further study revealed that *E. coli* infection upregulated the expression of Fpr2 in colon epithelial cells (Fig. 4E). Conversely, *E. coli* O22:H8 produced Fpr2 ligands that stimulate migration and growth of colon epithelial cells. In vitro experiments showed that the supernatant from *E. coli* O22:H8 induced migration of CT26 mouse colon epithelial cells in an Fpr2-dependent manner, as the activity of the *E. coli* supernatant was inhibited by Fpr2 antagonists WRW4 and BOC2 (Fig. 4F–G). Additionally, *E. coli* supernatant promoted faster closure of CT26 epithelial cell monolayer wounds, and this effect was attenuated by BOC2 (Fig. 4H). These results clearly demonstrate that commensal *E. coli* not only upregulates Fpr2 expression but also produces Fpr2 ligands that can enhance the migration and proliferation of colon epithelial cells through Fpr2.

### Fpr2 deficiency delayed the recovery of damaged colon mucosa of mice with colitis

**Fig. 5:**
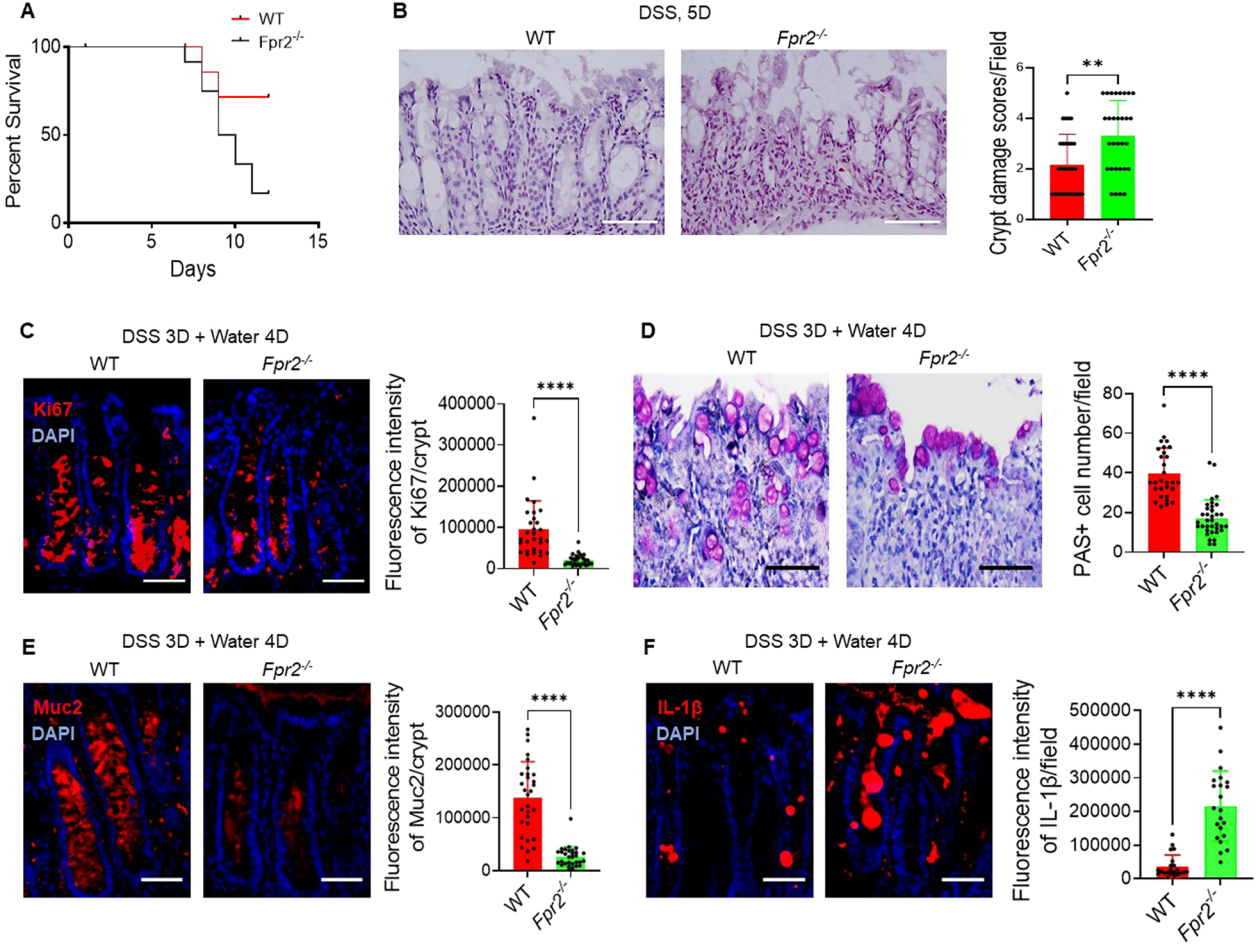
Fpr2 deficiency delayed the recovery of the damaged colon mucosa in mice with colitis. **A** Reduced survival of *Fpr2*^*−/−*^ mice after administration of 3% DSS in drinking water for 5 days followed by an additional 7 days of normal water intake. n = 12 mice/group. **B** Increased damage for colon mucosa in *Fpr2*^*−/−*^ mice treated with DSS for 5 days. Scale bar = 50 µm. Right: Quantitation of crypt damage in *Fpr2*^*−/−*^ mice after DSS treatment. n = 6 mice per group. **P < 0.01. **C** Reduced number of Ki67^+^ cells in the colon mucosa of *Fpr2*^*−/−*^ mice after administration of 3% DSS in drinking water for 3 days followed by an additional 4 days of normal water intake. Scale bar = 30 µm. Right: Quantitation of Ki67^+^ cells per crypt, n = 30 crypts from 4 mice/group. ****P < 0.0001. **D** Reduced number of goblet cells in the colon of *Fpr2*^-/-^ mice. An alcian blue stain. Scale bar = 50 μm. Right panel: Quantitation of PAS+ goblet cell number in the colon, n = 29-35 fields from 4 mice/group. ****P < 0.0001. **F** Increased production of IL-1β in the colon mucosa of *Fpr2*^*−/−*^ mice after administration of 3% DSS in drinking water for 3 days followed by an additional 4 days of normal water intake. Red: IL-1β, Blue: DAPI. Scale bar = 30 μm. Right: Quantitative IL-1β + fluorescence intensity, n = 30 crypts from 4 mice/group. ****P < 0.0001

We induced acute colitis in WT and *Fpr2*^*−/−*^ mice by administering 5% dextran sulfate sodium (DSS) in drinking water, by day 12 post DSS intake, 83.3% *Fpr2*^*−/−*^ mice (10/12) died, while only 28.6% WT mice (4/14) died (Fig. 5A). Histologically, the colon epithelia of *Fpr2*^*−/−*^ mice were significantly damaged by day 5 and infiltrated by numerous leukocytes, whereas the colon epithelia of WT mice remained mostly intact (Fig. 5B). These findings indicate that *Fpr2*^*−/−*^ mice are highly sensitive to chemically induced colitis.

To investigate the role of Fpr2 in the recovery of damaged colon mucosa, the mice were administered 5% DSS for 3 days followed by normal drinking water for 4 days. The fluorescence intensity of Ki67 in colon crypt was significantly reduced in *Fpr2*^*−/−*^ mice compared to WT mice (Fig. 5C). Furthermore, the numbers of PAS + goblet cells (Fig. 5D) and the production of Muc2 (Fig. 5E) were significantly lower in the colon mucosa of *Fpr2*^*−/−*^ mice compared to WT mice. Additionally, the level of IL-1β was increased in the colon mucosa of *Fpr2*^*−/−*^ mice as compared to WT mice (Fig. 5F). These results indicate that Fpr2 deficiency impairs the repair process and increases inflammatory responses in the colon mucosa of mice with colitis.

### Fpr2 deficiency increased *E. coli* population in the colon of mice with colitis.

**Fig. 6:**
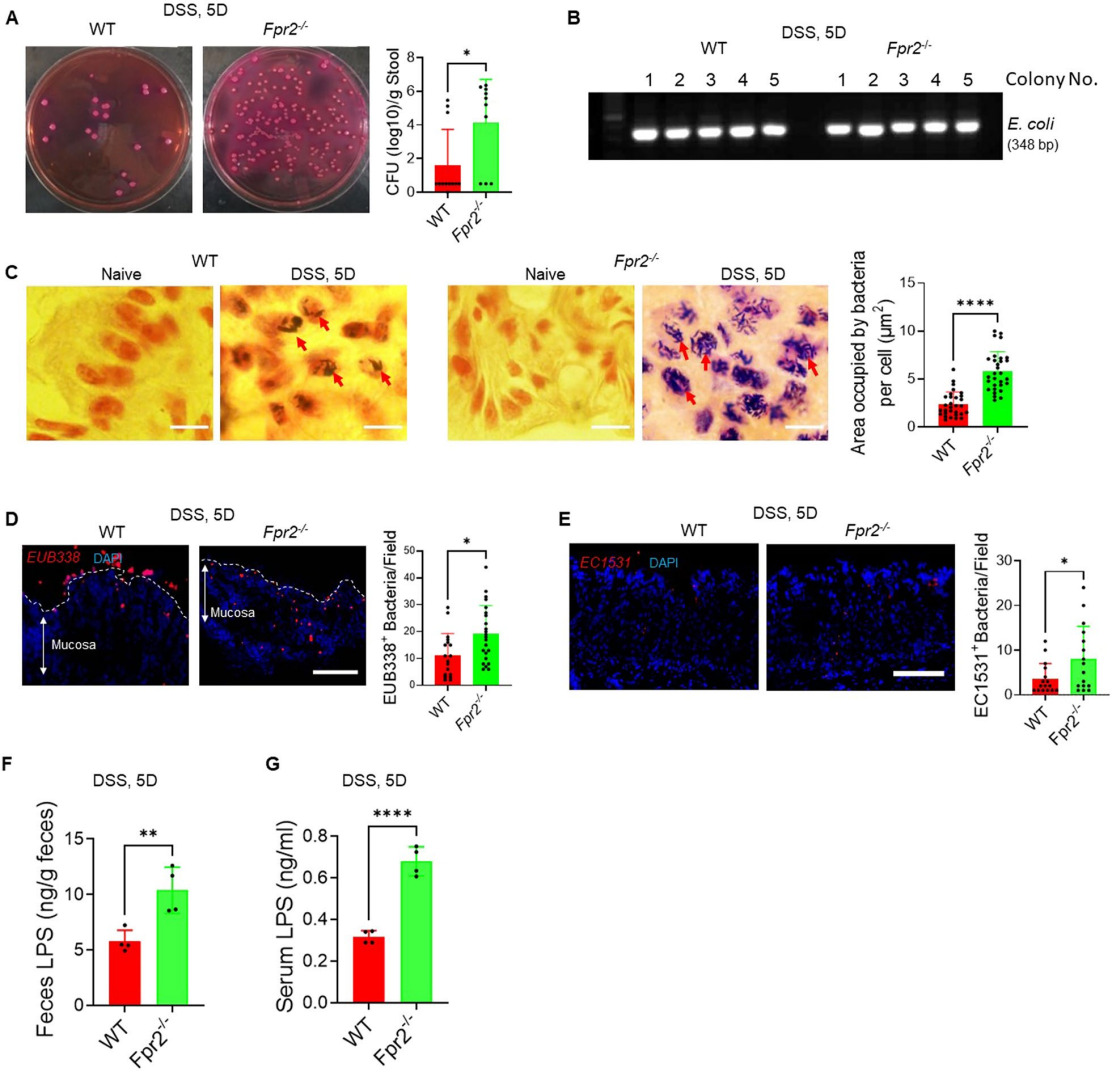
Fpr2 deficiency increased *E. coli* population in the colon of mice with colitis. **A** Increased *E. coli* counts in the feces of *Fpr2*^*−/−*^ mice after administration of 3% DSS in drinking water for 5 days. Right: Quantitation of *E. coli* in mouse feces. The results are expressed as *E. coli* CFU (Log10)/g feces. n = 10–12 mice/group. *P < 0.05. **B** PCR analysis confirmed that the bacteria isolated from the mouse feces were *E. coli*. **C.** Increased numbers of bacteria invaded the colon epithelial cells of *Fpr2*^*−/−*^ mice. Scale bar = 5 μm. Red arrows: Bacteria in colon epithelial cells. Right: Quantitation of bacteria in each epithelial cells. The results were expressed as area occupied by bacteria per cell (µm^2^). n = 30 cells/group, ****P < 0.0001. **D** FISH analysis showed that increased number of EUB338 + bacteria attached to the colon mucosa of *Fpr2*^*−/−*^ mice. Red: EUB338 probe^+^ bacteria, Blue: Nuclei. Scale bar = 30 μm. Right: Quantitation of the EUB338 probe^+^ bacteria/field. n = 18–23 fields from 5 mice per group, *P < 0.05. **E** FISH analysis showed that increased number of number of EC1531 probe^+^ *E. coli* attached to the colon mucosa of *Fpr2*^*−/−*^ mice. Red: EC1531^+^ *E. coli*, Blue: Nuclei. Scale bar = 30 μm. Right: Quantitation of the percentage (%) of EC1531^+^ *E. coli* in EUB338^+^ bacteria, n = 16 fields from 5 mice per group, *P < 0.05. **F** Increased level of LPS in the feces of *Fpr2*^*−/−*^ mice. The results were expressed as ng/g feces. n = 4 mice/group, **P < 0.01. **G** Increased level of LPS in the serum of *Fpr2*^*−/−*^ mice. The results were expressed as ng/ml serum. n = 4 mice/group, ****P < 0.0001

The mice were administered 5% DSS for 5 days, and subsequently, the feces were cultured on Violet Red Bile Lactose agar (VRBL)*.* The colony-forming units (CFU) of bacterial colonies grown on VRBL were quantified. The results revealed a significantly increase in bacterial CFU counts in the feces of *Fpr2*^*−/−*^ mice as compared to WT mice (Fig. 6A). Single colonies on the plates of VRBL were harvested and amplificated in LB Broth. The amplificated bacteria were then used to extract template DNA and subjected to PCR using primer specific for *E. coli,* allowing for the identification of *E. coli* colonies (Fig. 6B). The identified *E. coli* colonies were further cultured on VRBL plates, and single colonies were selected for 16S rRNA gene sequencing to confirm their classification as *E. coli* (Additional file 1: Fig. S2A, B).

Further studies revealed that the number of bacteria invading the colon epithelial cells was significantly increased in *Fpr2*^*−/−*^ mice with colitis (Fig. 6C). In fluorescence situ hybridization (FISH) using a fluorescence-labeled EUB338 probe, which targets most bacterial strains, demonstrated a significant increase in the number of bacteria adhering to the colon mucosa in *Fpr2*^−/−^ mice (Fig. 6D). Furthermore, FISH using an EC1531 probe, specifically targeting *E. coli,* showed an elevated number of *E. coli* adhering to the colon mucosa of *Fpr2*^*−/−*^ mice with colitis (Fig. 6E). The levels of lipopolysaccharide (LPS) were significantly increased in the feces (Fig. 6F) and serum (Fig. 6G) of *Fpr2*^*−/−*^ mice as compared to WT mice. Taken together, these results clearly indicate that Fpr2 deficiency leads to an increased presence of *E. coli* in the colon, suggesting that commensal *E. coli* may exhibit pathogenic characteristics under Fpr2 deficiency.

## Discussion

The microenvironment within the colon mucosa plays a crucial role in maintaining the balance of the gut microbiota. In this study, various defects associated with Fpr2 deficiency were observed, including shortened colon crypts, reduced production of Muc2 and antimicrobial peptides by epithelial cells, increased production of the pro-inflammatory cytokine IL-1β in the colon mucosa of naïve mice, and gut dysbiosis. There is mounting evidence to suggest that dysbiosis, characterized by an imbalance in the gut microbiota, is closely associated with inflammatory bowel disease (IBD) [5, 37]. Dysbiosis patterns commonly observed in IBD patients are characterized by a reduction in the diversity of commensal bacteria, particularly Firmicutes, and a relative increase in species belonging to Enterobacteriaceae [5, 38–40]. Multiple factors can disrupt the beneficial members of the gut microbiome, including antibiotic use, psychological and physical stress, radiation, altered gut peristalsis, and dietary changes [41]. Genetic deficiencies, such as mutations in the nucleotide-binding oligomerization domain-containing protein 2 (NOD2), have also been observed to result in gut dysbiosis in patients [42–48]. Muc2, serving as a primary barrier between the gut microbiome and the intestinal epithelium, plays a crucial role in maintaining gut homeostasis [49] and *Muc2*^*−/−*^ mice develop severe colitis due to the absence of protective mucous layers [50]**.** Similarly, deficiencies in cathelin-related antimicrobial peptide (CRAMP), reduced expression of β-defensin 2, and alterations in the gut homeostatic protein Fam3D have also been shown to disrupt the balance of the gut microbiota [29, 51–55]. In this study, we demonstrate that Fpr2 is essential for inducing epithelial cell growth and maturation, leading to the release of Muc2 and antimicrobial peptides, thereby maintaining the homeostasis of the colon mucosal microenvironment.

Enterobacterial blooms are frequently observed in cases of gut dysbiosis [3]. *E. coli,* a member of *Enterobacteriaceae* family, Proteobacteria phylum*,* typically represents a minor fraction of the microbiome in a healthy human colon [3, 56]**.** However, an elevated presence of *E. coli,* particularly mucosa-associated genotoxin-positive *E. coli,* has been consistently found in a significantly higher proportion of patients with Crohn's disease (CD) or ulcerative colitis (UC), two forms of inflammatory bowel disease (IBD) [38, 57–64]**.** In mouse models, deficiencies in CRAMP and reduced expression of β-defensin 2 have been shown to disrupt the balance of the gut microbiota, resulting in the overgrowth of *E. coli* in the colon [29, 51–54]**.** Histological analysis of human tumor samples has revealed extensive infiltration of inflammatory cells in pks-positive *E. coli*-infected HCT116 tumors [65], and the growth of tumors has been shown to be enhanced by colibactin-producing *E. coli* in xenograft and AOM/DSS-induced tumor models [66]**.** Therefore, an increase in *E. coli* counts serves as a significant indication of colon dysbiosis with potentially harmful consequences [4, 67]**.**

Recently, there has been significant attention given to the role of normal intestinal flora, including *E. coli*, in the recovery from colitis [68–71]. *E. coli* has been shown to promote the healing of colitis-related mucosal damage in the colon through the activation of the TLR4/NF-κB signaling pathway [68]. Additionally, *E. coli* has been found to protect mice from *Citrobacter rodentium* infection and DSS-induced colitis [69]. In our previous study, we demonstrated the expression of Fpr2 on mouse colonic epithelial cells, which can be activated by fMLF, a chemotactic peptide ligand derived from *E. coli*. We observed that the intake of DSS increased the expression of Fpr2 in both immature and mature epithelial cells of colonic crypts, suggesting the importance of Fpr2 in enabling epithelial cells to respond to locally and systemically available ligands in pathological conditions. *E. coli* O22:H8 produces ligands for both Fpr1 and Fpr2, which induce inflammatory cell migration [9] and promote the restoration of chemically induced mucosal damage [68]. In the current study, we identified two *E. coli* serotypes, O22:H8 and O91:H21, through whole genome sequencing. *E. coli* O22:H8 was present in the feces of both WT and *Fpr2*^*−/−*^ mice and, as a commensal bacterium, did not cause significant damage to colon epithelial cells compared to *E. coli* O91:H21, both in vitro and in vivo.

Infection with *E. coli* O22:H8 stimulated the upregulation of Fpr2 expression, and its products induced the migration and proliferation of colon epithelial cells through Fpr2. However, Fpr2 deficiency led to an increased population of *E. coli* in the mouse colon and delayed the recovery of damaged colon epithelial cells, indicating the involvement of Fpr2 expression in the effects of commensal *E. coli*. Nonetheless, the overgrowth of *E. coli* O22:H8 observed in *Fpr2*^*−/−*^ mice has pathological significance, such as a significant reduction in inflammatory cell accumulation at *E. coli*-infected sites and increased mortality in *Fpr2*^*−/−*^ mice [9]. Thus, the Fpr2 deficiency-induced overgrowth of *E. coli* O22:H8 appears to be harmful to the host.

In contrast to *E. coli* O22:H8, *E. coli* O91:H21 represents a relatively small proportion of *E. coli* colonies isolated from the mouse feces in our study. It has been reported that *E. coli* O91:H21 can be found in food, animals, or the environment and may cause severe diseases, including hemolytic-uremic syndrome [36]. *E. coli* O91:H21 consists of many strains, some of which carry stx genes [36]. Both O91:H21 and O22:H8 belong to the group of Shiga toxin-producing *E. coli* (STEC) strains and express long polar fimbriae (lpfA) [36], which is a potential adherence factor originally described in *Salmonella* [72, 73]. Four genetic variants of lpfA, namely lpfA (O157/OI-141), lpfA (O157/OI-154), lpfA (O26), and lpfA (O113), have been identified in Shiga toxin-producing *E. coli* (STEC) [74]. LpfA is associated with the adherence and invasion capacity of bacteria to epithelial cells [75], and its expression in *E. coli* strains is important for pathogenicity [35, 76]. In our study, the gene for Long Polar Fimbriae Type I (lpfA), a recognized marker for virulent isolates of pathogenic *E. coli*, is more readily amplified by PCR in *E. coli* O91:H21 compared to the O22:H8 strain, although whole genome sequencing results revealed that both *E. coli* O91:H21 and *E. coli* O22:H8 have the same number of base pairs (in PPM) for the lpfA gene. When comparing symptomatic strains of STEC O91:H21, such as ATCC 51435 and ATCC 51434, which are known to be highly virulent in an experimental infection mouse model, with asymptomatic strains of STEC O91:H21 isolated from a STEC outbreak in Korea, the asymptomatic STEC O91:H21 isolates exhibited a significantly reduced adherence phenotype and cytopathic effects due to the transcriptional repression of the genes encoding type-1 fimbriae in the asymptomatic isolates [77, 78]. This suggests the importance of fimbriae for *E. coli* in adhering to and invading colon epithelial cells. Furthermore, analyses using the Virulence Factor Database (Vfdb) [34] suggest the presence of three virulence factors in O91:H21 but not in O22:H8, which are produced by a generic Type II secretory machinery associated with enterotoxicity in *E. coli* [79]. Our studies in GF mice clearly demonstrate the more proliferative and invasive nature of *E. coli* O91:H21 in the colon. However, further investigation is required to determine the origin of *E. coli* O91:H21 in mice and whether the isolate we obtained belongs to a mutated variant of the originally harmless strain.

## Conclusions

Fpr2 is highly expressed in innate immune cells and non-hematopoietic cells, including epithelial cells. Fpr2 is required to induce epithelial cell growth and maturation, releasing Muc2 and antimicrobial peptides, thereby maintaining the homeostasis of the colon mucosal microenvironment and the balance of gut microbiota. Fpr2 deficiency results in gut dysbiosis and an increased population of *E. coli* in the colon, one of the common bacteria, which is an important indication of colon dysbiosis with detrimental consequences [4, 67]**.** Fpr2 is required to interact with *E. coli* derived Fpr2 ligands, stimulating colon epithelial cell regeneration. *E. coli* infection induces the upregulation of Fpr2 expression, which interacts with endogenous Fpr2 ligands such as CRAMP to stimulate colon epithelial cell proliferation [29]. Fpr2 deficiency, resulting in an increase of *E. coli* in the colon of *Fpr2*^*−/−*^ mice with colitis, may be a compensatory response by the host. The importance of Fpr2 in the maintenance of host homeostasis and the control of inflammation and inflammation-related diseases is increasingly recognized, providing an opportunity for developing a new arm of pharmacological targeting. Understanding how Fpr2 operates in a cell-specific manner should guide the development of new therapeutics for human IBD.

### Supplementary Information

**Additional file 1: Figure S1.** Impaired colon mucosal barrier in *Fpr2*^*-/-*^ mice. **A.** Reduced CRAMP production in colon crypts of *Fpr2*^*-/-*^ mice. Red: CRAMP, Blue: Nuclei. Scale bar = 50 μm. **Right panel:** Quantitation of CRAMP^+^ fluorescence intensity, n = 18-20 crypts from 6 mice/group, ***P < 0.001. **B.** Reduced CRAMP level in the feces of *Fpr2*^*-/-*^ mice. The concentration of CRAMP was expressed as pg/1 mg protein in stool. n = 8 mice/group, *P < 0.05. **C.** Reduced production of β-Defensin 2 in the colon crypts of *Fpr2*^*-/-*^ mice. Red: β-Defensin 2, Blue: Nuclei. Scale bar = 50 μm. **Right panel:** Quantitation of β-Defensin 2^+^ fluorescence intensity, n = 20 crypts from 6 mice/group, ***P < 0.001. **D.** Reduced β-Defensin 2 level in the feces of *Fpr2*^*-/-*^ mice. The concentration of β-Defensin 2 was expressed as pg/1 mg protein in stool. n = 8 mice/group, *P < 0.05. **Figure S2.** 16S rRNA gene sequencing. **A.** The result of 16S rRNA gene sequencing for *E. coli* isolated with Violet red bile lactose agarin this study. **B.** Comparison of 16S rRNA gene sequencing of *E. coli *isolated from VRBL in this study to that of other *E. coli* published in online. **Figure S3.** Comparison of 16S rRNA gene sequence of two different *E. coli* strains isolated from mouse feces. A small number of colonies of *E. coli* displayed differences in nucleotides #52, 113, 116, 136, 263, 293 and 424 as compared to most *E. coli* colonies. **Figure S4.** Similar phonotypes between two *E. coli* strains isolated from mouse feces. **A**. Similar results of Gram Staining for* E. coli *Type I* and *Type II. *E. coli *smear was stained with Gram Stain Kit. Red: Gram negative bacteria. Scale bar = 5 μm. **B. **Similar results of FISH with EC1531 probe for *E. coli-*Type I* and *Type II. *E. coli *smear was obtained by *in situ* hybridization with EC1531 probe conjugated to CY3. Red: EC1531^+^ bacteria. Scale bar = 5 μm. **C. **Similar results of PCR for *E. coli-*Type I* and *Type II. Colonies of *E. coli *from Type I and II were cultured in VRBL, respectively. Single colonies were then selected to expand in LB for 24 h and DNA was isolated followed by PCR amplification with *E. coli *primers.

## Data Availability

All data generated or analyzed during this study are included in this manuscript. 16S rRNA gene amplicon sequence data for *E. coli* was deposited in Science Data Bank, https://doi.org/10.57760/sciencedb.j00111.00005. Whole genome sequencing data for *E. coli* O22:H8 and O91H21 were deposited in NCBI GenBank (ID: SUB11613054) and are available from the corresponding author upon request.

## Abbreviations

CD: Crohn’s disease
CFU: Colony-forming unit
CRAMP: Cathelin-related Antimicrobial Peptide
DAPI: 6-Diamidino-2-phenylindole
FPR2: Formyl peptide receptor 2
fMLF: *N*‐Formyl‐methionyl‐leucyl‐phenylalanine
FISH: Fluorescence in situ hybridization
GF: Germ-free
GPCRs: G-protein-coupled chemoattractant receptors
IBD: Inflammatory bowel disease
LPS: Lipopolysaccharides
lpfA: Long Polar Fimbriae Type I
UC: Ulcerative colitis
