# Correction: The beneficial effects of commensal *E. coli* for colon epithelial cell recovery are related with Formyl peptide receptor 2 (Fpr2) in epithelial cells

**DOI:** 10.1186/s13099-023-00585-6

**Published:** 2023-11-30

**Authors:** Keqiang Chen, John McCulloch, Rodrigo Das Neves, Gisele Rodrigues, Wang-Ting Hsieh, Wanghua Gong, Teizo Yoshimura, Jiaqiang Huang, Colm O’hUigin, Simone Diflippantonio, Matthew McCollum, Georgette Jones, Scott K. Durum, Giorgio Trinchieri, Ji Ming Wang

**Affiliations:** 1 Laboratory of Cancer Innovation, Center for Cancer Research, National Cancer Institute at Frederick, Frederick, MD 21702 USA; 2 Laboratory of Integrative Cancer Immunology, Center for Cancer Research, National Cancer Institute, Bethesda, MD 20892 USA; 3 Animal Health Diagnostic Laboratory, Frederick National Laboratory for Cancer Research, Frederick, MD 21702 USA; 4 Basic Research Program, Leidos Biomedical Research, Inc, Frederick, MD 21702 USA; 5 Department of Pathology and Experimental Medicine, Graduate School of Medicine, Dentistry and Pharmaceutical Sciences, Okayama University, Okayama, 700‑8558 Japan; 6 College of Life Sciences, Beijing Jiaotong University, Beijing, 100044 People’s Republic of China; 7 Gnotobiotics Facility, Frederick National Laboratory for Cancer Research, Frederick, MD 21702 USA

**Correction** **:** **Gut Pathogens (2023) 15:28** **https://doi.org/10.1186/s13099-023-00557-w**

In this article [1] the author name Gisele Rodrigues was incorrectly written as Gisele Roderigues.

The original article has been corrected.
