# Supplementary material for: The beneficial effects of commensal E. coli for colon epithelial cell recovery are related with Formyl peptide receptor 2 (Fpr2) in epithelial cells
Source: Gut Pathog. 2023 Jun 15;15:28. doi: 10.1186/s13099-023-00557-w (PMC10268441; doi:10.1186/s13099-023-00557-w)
Supplement: Supplementary file 1 — Additional file 1: Figure S1. Impaired colon mucosal barrier in Fpr2-/- mice. A. Reduced CRAMP production in colon crypts of Fpr2-/- mice. Red: CRAMP, Blue: Nuclei. Scale bar = 50 μm. Right panel: Quantitation of CRAMP+ fluorescence intensity, n = 18-20 crypts from 6 mice/group, ***P < 0.001. B. Reduced CRAMP level in the feces of Fpr2-/- mice. The concentration of CRAMP was expressed as pg/1 mg protein in stool. n = 8 mice/group, *P < 0.05. C. Reduced production of β-Defensin 2 in the colon crypts of Fpr2-/- mice. Red: β-Defensin 2, Blue: Nuclei. Scale bar = 50 μm. Right panel: Quantitation of β-Defensin 2+ fluorescence intensity, n = 20 crypts from 6 mice/group, ***P < 0.001. D. Reduced β-Defensin 2 level in the feces of Fpr2-/- mice. The concentration of β-Defensin 2 was expressed as pg/1 mg protein in stool. n = 8 mice/group, *P < 0.05. Figure S2. 16S rRNA gene sequencing. A. The result of 16S rRNA gene sequencing for E. coli isolated with Violet red bile lactose agarin this study. B. Comparison of 16S rRNA gene sequencing of E. coli isolated from VRBL in this study to that of other E. coli published in online. Figure S3. Comparison of 16S rRNA gene sequence of two different E. coli strains isolated from mouse feces. A small number of colonies of E. coli displayed differences in nucleotides #52, 113, 116, 136, 263, 293 and 424 as compared to most E. coli colonies. Figure S4. Similar phonotypes between two E. coli strains isolated from mouse feces. A. Similar results of Gram Staining for E. coli Type I and Type II. E. coli smear was stained with Gram Stain Kit. Red: Gram negative bacteria. Scale bar = 5 μm. B. Similar results of FISH with EC1531 probe for E. coli-Type I and Type II. E. coli smear was obtained by in situ hybridization with EC1531 probe conjugated to CY3. Red: EC1531+ bacteria. Scale bar = 5 μm. C. Similar results of PCR for E. coli-Type I and Type II. Colonies of E. coli from Type I and II were cultured in VRBL, respectively. Single colonies were [file 13099_2023_557_MOESM1_ESM.doc]

**Additional file**

**The beneficial effects of commensal *E. coli* for colon epithelial cell recovery are related with Formyl peptide receptor 2 (Fpr2) in epithelial cells**

Keqiang Chen, John McCulloch, Rodrigo Das Neves, Gisele Roderigues, Wang-Ting Hsieh, Wanghua Gong, Teizo Yoshimura, Jiaqiang Huang, Colm O’hUigin, Simone Difilippantonio, Matthew McCollum, Georgette Jones, Scott K. Durum, Giorgio Trinchieri, and Ji Ming Wang


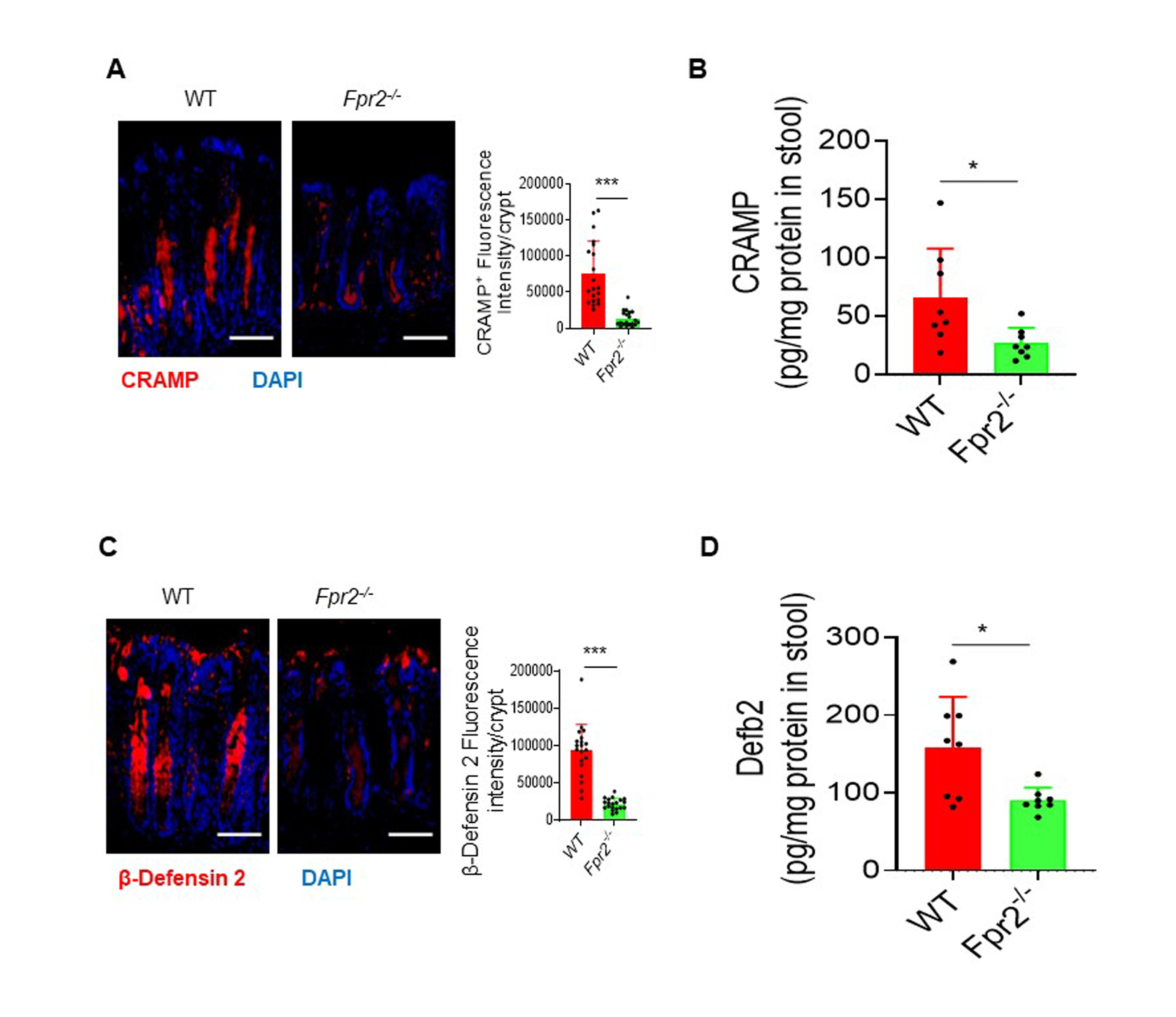


**Figure S1.** **Impaired colon mucosal barrier in *Fpr2^-/-^* mice. A.** Reduced CRAMP production in colon crypts of *Fpr2^-/-^* mice. Red: CRAMP, Blue: Nuclei. Scale bar = 50 μm. **Right panel:** Quantitation of CRAMP^+^ fluorescence intensity, n = 18-20 crypts from 6 mice/group, ****P* < 0.001. **B.** Reduced CRAMP level in the feces of *Fpr2^-/-^* mice. The concentration of CRAMP was expressed as pg/1 mg protein in stool. n = 8 mice/group, **P* < 0.05. **C.** Reduced production of β-Defensin 2 in the colon crypts of *Fpr2^-/-^* mice. Red: β-Defensin 2, Blue: Nuclei. Scale bar = 50 μm. **Right panel:** Quantitation of β-Defensin 2^+^ fluorescence intensity, n = 20 crypts from 6 mice/group, ****P* < 0.001. **D.** Reduced β-Defensin 2 level in the feces of *Fpr2^-/-^* mice. The concentration of β-Defensin 2 was expressed as pg/1 mg protein in stool. n = 8 mice/group, **P* < 0.05.


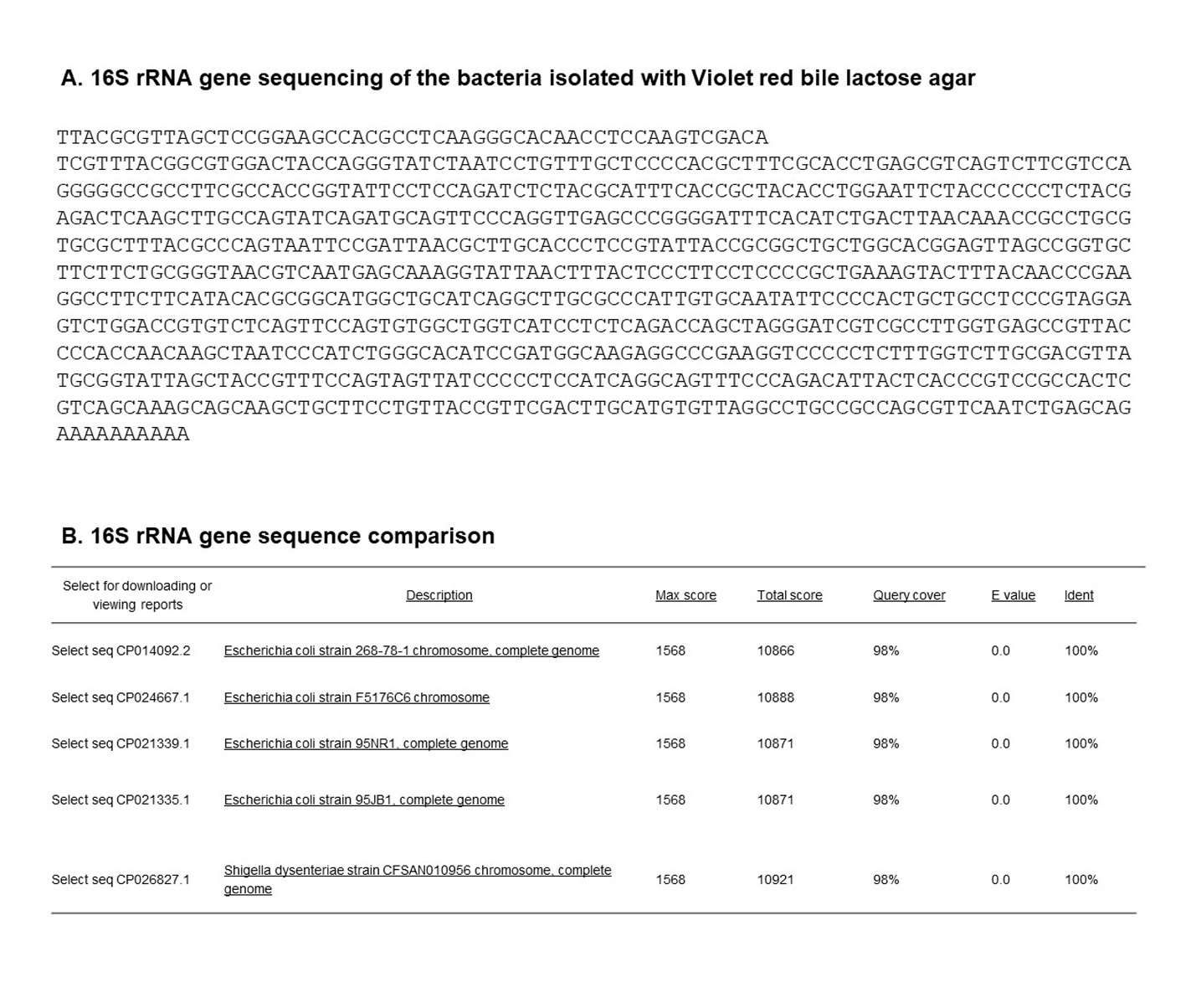


**Figure S2. 16S rRNA gene sequencing. A.** The result of 16S rRNA gene sequencing for *E. coli* isolated with Violet red bile lactose agar (VRBL) in this study. **B.** Comparison of 16S rRNA gene sequencing of *E. coli* isolated from VRBL in this study to that of other *E. coli* published in online.


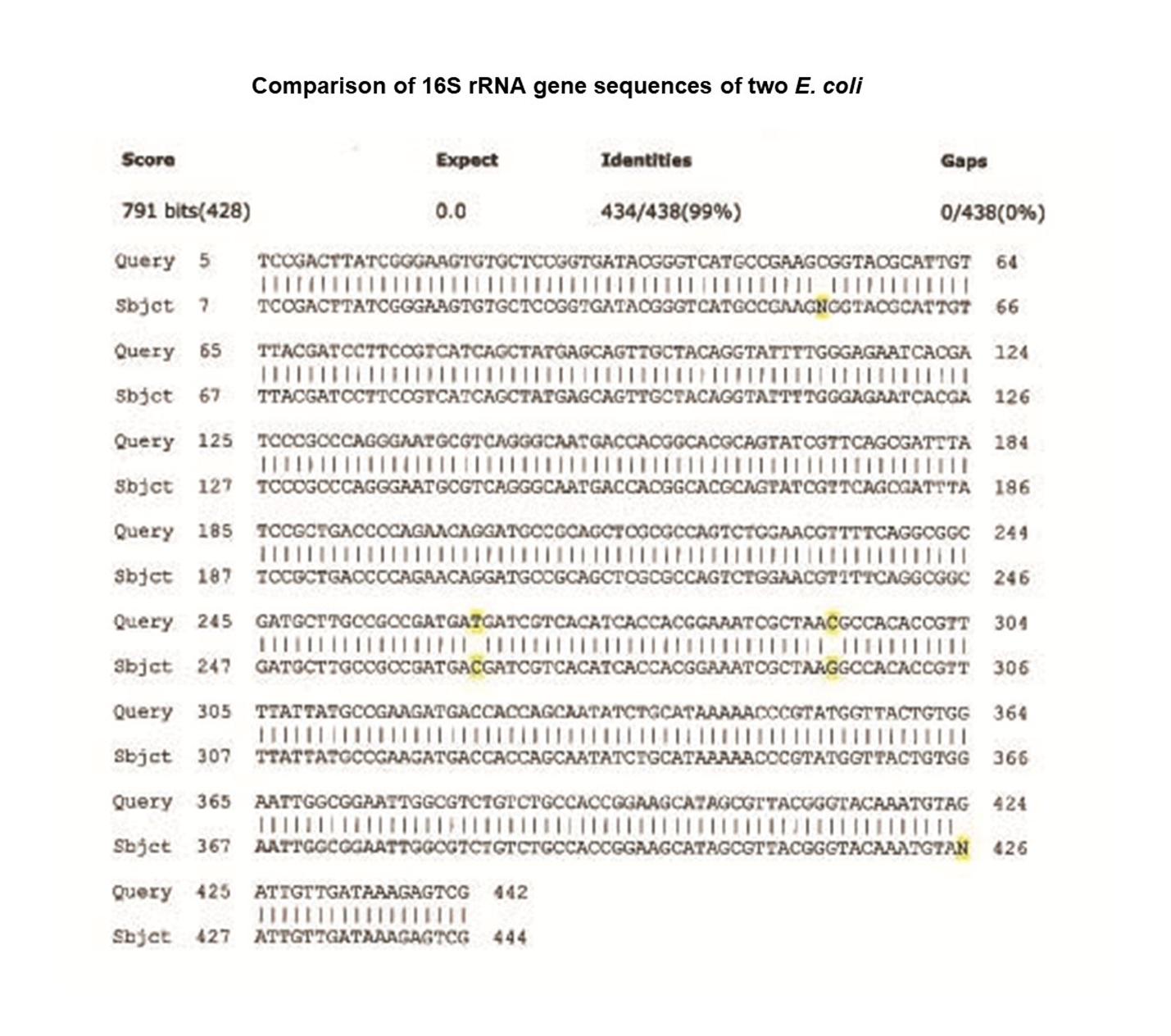


**Figure S3. Comparison of 16S rRNA gene sequence of two different *E. coli* strains isolated from mouse feces.** A small number of colonies of *E. coli* displayed differences in nucleotides #52, 113, 116, 136, 263, 293 and 424 as compared to most *E. coli* colonies

**
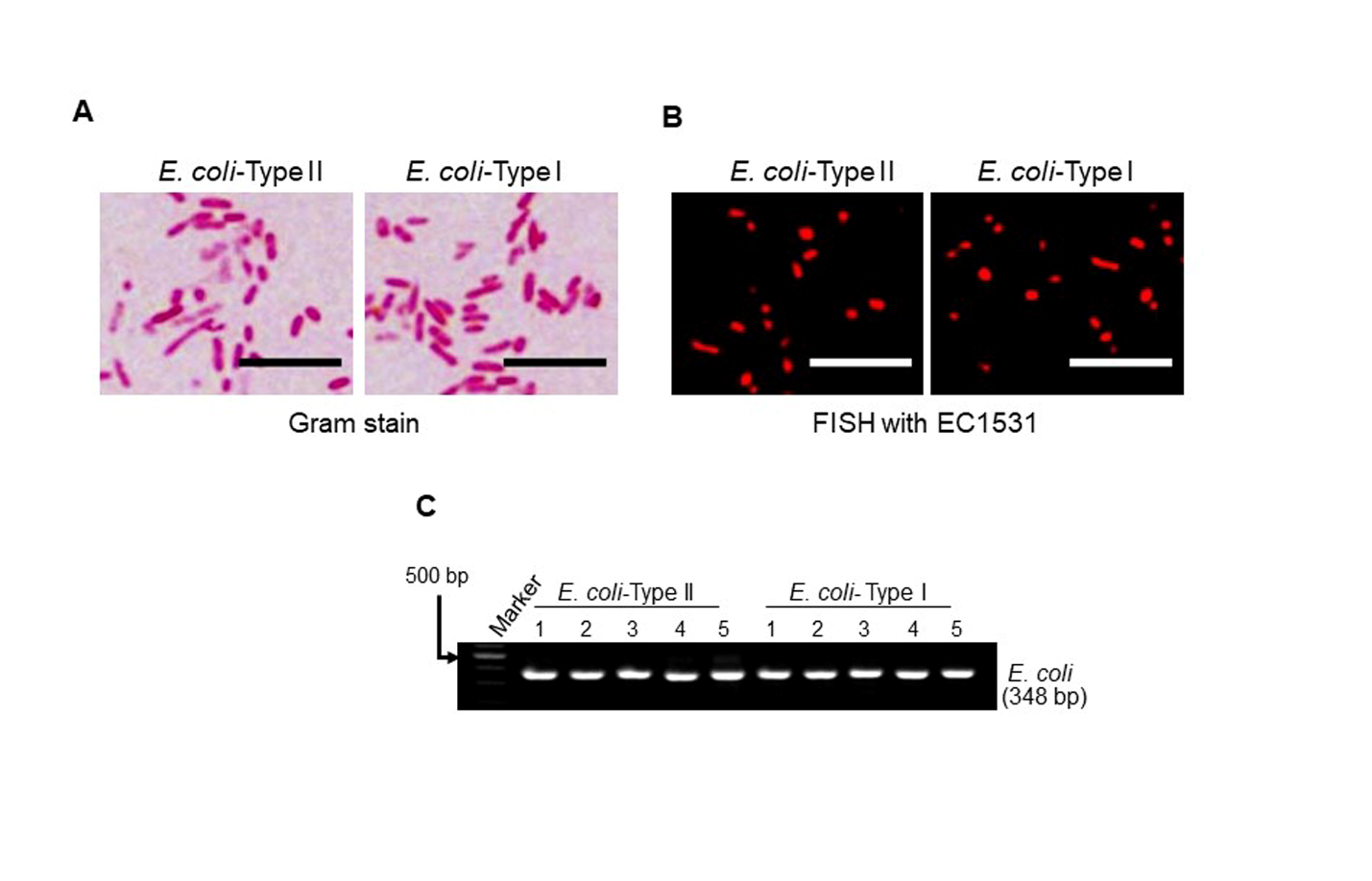
**

**Figure S4. Similar phonotypes between two E. coli strains isolated from mouse feces. A.** Similar results of Gram Staining for *E. coli* Type I *and* Type II. *E. coli* smear was stained with Gram Stain Kit. Red: Gram negative bacteria. Scale bar = 5 μm. **B.** Similar results of FISH with EC1531 probe for *E. coli-*Type I *and* Type II. *E. coli* smear was obtained by *in situ* hybridization with EC1531 probe conjugated to CY3. Red: EC1531^+^ bacteria. Scale bar = 5 μm. **C.** Similar results of PCR for *E. coli-*Type I *and* Type II. Colonies of *E. coli* from Type I and II were cultured in VRBL, respectively. Single colonies were then selected to expand in LB for 24 h and DNA was isolated followed by PCR amplification with *E. coli* primers.
